# Machine Learning Classification of Prostate Cancer Genomic Sequences Using K-Mer and Sequence-Derived Features

**DOI:** 10.4236/cmb.2026.162002

**Published:** 2026-06-24

**Authors:** Kuldeep Rawat, Hirendra Nath Banerjee, Jamie Noble, Saa Naudia Deloatch, Satyendra Banerjee, Sachin Shetty, Soumya Banerjee

**Affiliations:** 1 Department of Mathematics, Computer Science, and Engineering Technology, Elizabeth City State University, Elizabeth City, USA; 2 Department of Natural Sciences, Elizabeth City State University, Elizabeth City, USA; 3 Virginia Modeling and Simulation Center, Old Dominion University, Suffolk, USA

**Keywords:** Prostate Cancer, DNA Sequence Classification, K-Mer Analysis, Sequence-Derived, Random Forest Algorithm, SMOTE, Machine Learning, Health Disparities

## Abstract

Prostate cancer disproportionately impacts African American men, who experience significantly higher mortality rates and earlier disease onset than other populations. Current diagnostic approaches, including prostate-specific antigen testing and biopsy, lack sufficient specificity and sensitivity, underscoring the need for accurate, molecular-level classification tools. This paper presents a machine learning framework for binary classification of genomic DNA sequences as cancerous or healthy. A dataset of 1684 FASTA-formatted sequences obtained from the National Library of Medicine - GenBank was analyzed, with 1662 sequences retained after quality control filtering. Feature engineering yielded 67 attributes, including GC content, Shannon entropy, sequence length, and trinucleotide k-mer frequencies. To address class imbalance, we applied the Synthetic Minority Over-sampling Technique to the training data. Seven classification algorithms were evaluated using stratified train–test splits, cross-validation, and hyperparameter optimization. Among the models, the optimized Random Forest classifier achieved superior performance, with a cross-validation accuracy of 97.2% (±0.006), a weighted F1-score of 0.95, a cancer-class recall of 0.96, and an ROC-AUC of 0.974. Feature importance analysis identified sequence length and Shannon entropy as the most discriminative predictors, followed by specific trinucleotide motifs (TTC, AAC, ACC, and GGG). These results demonstrate the potential of interpretable machine learning approaches for genomic sequence-based PCa classification, offering a promising pathway toward improved, equitable diagnostic tools for high-risk populations.

## 1. Introduction

Prostate cancer (PCa) is the most prevalent non-cutaneous malignancy among men in the United States, with an estimated 268,490 new cases projected for 2025 [1]. While incidence and outcomes have improved for many demographic groups, stark racial disparities persist. African-American (AA) men bear a disproportionate burden of disease, exhibiting the highest PCa incidence in the nation at 183.4 cases per 100,000, presenting on average two years earlier than White men, and facing a 2.2-fold greater risk of PCa-specific mortality [2]–[5]. AA men are also more likely to be diagnosed with aggressive, high-grade tumors and at later disease stages, resulting in fewer curative treatment options, higher morbidity, and excess mortality [6]–[8]. These disparities are multifactorial, encompassing socioeconomic barriers, systemic inequities in healthcare access, and potentially distinct tumor biology [3] [4].

The two most common clinical tools for PCa detection, prostate-specific antigen (PSA) testing and needle biopsy, carry significant limitations. PSA, the only blood-based biomarker in routine clinical use, has poor specificity, generating high rates of false positives that lead to unnecessary biopsies and patient anxiety [9] [10]. Biopsy-based Gleason grading, the standard method for assessing tumor aggressiveness, is inherently subjective, prone to inter-observer variability, and susceptible to sampling error, which can lead to the most aggressive tumor foci being missed [5]–[9]. These limitations underscore the urgent clinical need for objective, reproducible, and molecularly grounded diagnostic tools that operate independently of tissue morphology assessment.

Advances in computational genomics and machine learning (ML) offer a promising path toward sequence-based, objective PCa classification. DNA sequences encode rich biological information at the molecular level, and natural language processing (NLP)-inspired techniques have demonstrated substantial power for sequence analysis. K-mer frequency analysis, which decomposes DNA sequences into overlapping nucleotide substrings of fixed length k, captures local compositional patterns associated with gene regulation, repeat-element content, mutational signatures, and oncogene activation. These features are computationally inexpensive, biologically interpretable, and have been shown to discriminate against cancer from normal tissue across multiple cancer types [11] [12].

Despite growing interest in sequence-level ML for cancer diagnostics, few studies have systematically evaluated multiple classifier families on prostate-specific genomic sequence data, rigorously addressed class imbalance, or applied such frameworks with an explicit focus on health equity for African-American populations. The Artificial Intelligence/Machine Learning Consortium to Advance Health Equity and Researcher Diversity (AIM-AHEAD) program, funded by the National Institutes of Health (NIH), was established precisely to build AI and ML research capacity at Historically Black Colleges and Universities (HBCUs) and other minority-serving institutions, and to develop AI tools that address health disparities.

In this paper, we present a rigorous ML framework for binary classification of prostate genomic sequences developed through the AIM-AHEAD project at Elizabeth City State University (ECSU). The framework applies k-mer frequency feature engineering and systematic multi-classifier evaluation with SMOTE-based class balancing to distinguish cancerous from healthy prostate sequences sourced from the National Library of Medicine (NLM) GenBank. We report a complete experimental pipeline with quantitative performance benchmarks, feature importance analysis, and biologically grounded interpretation of discriminatory sequence motifs.

## 2. Related Work

Machine learning has been increasingly applied to PCa diagnosis, grading, and prognosis across multiple data modalities. Early computational studies employed gene expression profiling and mutational signatures to stratify patients by risk, achieving accuracy exceeding 90% in select cohorts [11] [12]. Deep learning models applied to digitized histopathological images, including convolutional neural networks, MobileNet, U-Net, and graph convolutional networks, have demonstrated Gleason grading performance approaching that of expert pathologists on whole-slide images [13]–[18]. While powerful, these imaging-based approaches require specialized digital pathology infrastructure and expert-annotated training data and do not leverage molecular sequence-level information, limiting their applicability in lower-resource clinical settings.

At the genomic sequence level, k-mer-based representations have been used to distinguish cancer from normal tissue across multiple cancer types [13]. The frequency of specific oligonucleotide motifs reflects codon usage bias, repeat-element composition, and regulatory-sequence architecture, all of which are systematically altered in cancer genomes through somatic mutations, copy-number changes, and epigenetic remodeling. Bag-of-Words k-mer models, combined with classical ML classifiers such as Random Forest and Support Vector Machines, have achieved competitive performance in sequence classification tasks with relatively low computational requirements compared to deep learning alternatives [19]–[21].

Despite this progress, important gaps remain. Few published frameworks have rigorously benchmarked multiple ML classifiers on prostate-specific genomic sequence data while explicitly correcting for class imbalance. Studies specifically targeting AA PCa genomics or employing frameworks designed with health equity in mind are particularly scarce. Our work addresses these gaps by presenting a systematic, interpretable ML pipeline with rigorous class-balancing and feature-importance analysis applied to prostate cancer sequence data.

## 3. Materials and Methods

### 3.1. Dataset Acquisition and Composition

Genomic sequence data were retrieved from the NLM GenBank repository. Prostate cancer patients’ genomic DNA sequences were downloaded in the FASTA format, along with gene annotations and GENBANK accession numbers, using NCBI search engine queries; healthy prostate genomic DNA sequences were also obtained using a similar method via a GENBANK query. Sequences were labeled as cancerous (label = 1) if the associated GenBank record contained one or more of the following terms in the DEFINITION, TITLE, or KEYWORDS fields: “prostate cancer,” “prostate carcinoma,” “prostatic adenocarcinoma,” or “PCa.” Sequences were labeled as healthy (label = 0) if the record contained “normal prostate,” “benign prostatic,” “healthy prostate,” or “non-cancerous prostate” in the same fields and contained no cancer-related terminology. Records with ambiguous or conflicting annotations were excluded.

The dataset comprised 1684 FASTA-format DNA sequences: 1544 annotated as cancerous (PCa-positive) and 140 annotated as healthy (non-cancerous prostate tissue). This class ratio of approximately 11:1 (cancerous: healthy) reflects the composition of available public repositories and necessitated class balancing before model training. All sequences originated from human prostate tissue.

Inclusion criteria required sequences to be of confirmed human prostate origin. Sequences with ambiguous nucleotide content, bases denoted by IUPAC ambiguity codes (N, R, Y, S, W, K, M, B, D, H, V), exceeding 5% of total sequence length were excluded to ensure feature quality and avoid spurious k-mer counts. Specifically, 21 cancerous sequences contained more than 5% N (undetermined) bases due to low sequencing quality, and one additional record was excluded because it contained non-DNA characters (protein sequence or annotation text inadvertently included in the FASTA file). Following quality control, 1662 sequences were retained for all downstream analyses (1522 cancerous, 140 healthy).

### 3.2. Sequence Preprocessing and Quality Control

All FASTA sequences underwent a standardized, reproducible preprocessing pipeline prior to feature extraction: (1) sequence parsing and header extraction using BioPython; (2) removal of sequences exceeding the 5% ambiguous nucleotide threshold; (3) conversion of all nucleotides to uppercase; and (4) removal of any remaining non-ATGC characters before feature computation, ensuring all downstream k-mer calculations were based on unambiguous nucleotides only.

### 3.3. Feature Engineering

#### 3.3.1. Physicochemical and Compositional Features

Three global sequence-level features were computed for each DNA sequence: 1) GC content, proportion of guanine and cytosine bases, associated with gene density, expression levels, and chromatin accessibility; 2) Shannon entropy (*H* = −∑ *p*_*i*_ log^2^ (*p*_*i*_)) over the four nucleotide frequencies, measuring nucleotide compositional diversity and informational complexity; and 3) sequence length in base pairs. These three features provide biologically meaningful, computationally inexpensive descriptors known to differ between cancerous and non-cancerous genomic regions.

#### 3.3.2. K-Mer Features

K-mer features were generated by decomposing each sequence into all overlapping nucleotide substrings of length k = 3 (trinucleotides). Trinucleotide k-mers were selected because: a) they span the biologically fundamental unit of the codon; b) the resulting feature space (4^3^ = 64 possible trinucleotides) remains tractable for classical ML classifiers without dimensionality reduction; and c) trinucleotide mutational signatures are well-established cancer genomics features linked to specific mutagenic processes [9] [11]. A Bag-of-Words frequency model was applied using scikit-learn’s CountVectorizer to convert k-mer occurrence counts into normalized frequency vectors. Combined with the three physicochemical features, the total feature dimensionality was 67 features per sequence.

For a sequence of length *L* containing *n(t)* occurrences of trinucleotide *t,* the normalized k-mer frequency is computed as: *f* (*t*) = *n*(*t*) (*L*−*k* +1), where (*L*−*k*+1) is the total number of overlapping k-mers of length *k* = 3 extractable from the sequence. This normalizes counts by the number of possible k-mer positions rather than by total nucleotide count, ensuring that frequency estimates are comparable across sequences of different lengths. Sequences shorter than 10 bp were flagged during quality control because fewer than 8 overlapping trinucleotides can be extracted, making frequency estimates statistically unreliable. In practice, the minimum sequence length in the post-QC dataset was 10 bp (one cancerous sequence); all healthy sequences were at least 31 bp in length. We note that the 10 bp minimum boundary is conservative and that sequences this short are present only in the cancerous class, where they likely represent short genomic fragments or partial submissions. We have added a note to the Limitations section acknowledging that k-mer frequency estimates derived from sequences shorter than approximately 50 bp should be interpreted with caution, as the effective sample size of k-mer observations is small and estimates are subject to higher variance.

#### 3.3.3. Class Balancing via SMOTE

The binary classification dataset exhibited a substantial class imbalance, with cancerous sequences outnumbering healthy sequences by approximately 11:1. To address this, the Synthetic Minority Over-sampling Technique (SMOTE) [22] was applied exclusively to the training split (after train/test partitioning and before classifier training), generating synthetic feature vectors for the healthy (minority) class by interpolating between existing minority-class samples in feature space. SMOTE was strictly not applied to the held-out test set, preserving the natural class distribution for unbiased performance evaluation.

### 3.4. Model Development and Evaluation

Seven ML classifiers spanning a range of algorithmic families (both linear and non-linear) were evaluated: Logistic Regression (LR), Linear Discriminant Analysis (LDA), K-Nearest Neighbors (KNN), Decision Tree (DT), Support Vector Classifier (SVC), Random Forest (RF), and Gradient Boosting Machine (GBM). The dataset was partitioned into 80% for training and 20% for testing using stratified random sampling. Because sequences were retrieved from GenBank based on organism and tissue annotation rather than from a patient-linked clinical database, patient-level identifiers are not systematically available in the metadata. As a result, we cannot guarantee that sequences originating from the same patient, the same tissue sample, or the same GenBank submission are confined exclusively to either the training or test set. If multiple sequences share a biological source, their appearance in both splits would constitute a form of data leakage that could inflate reported test performance by allowing the classifier to implicitly recognize source-specific sequence characteristics rather than general cancer-associated features.

Model selection and hyperparameter optimization were conducted using three-fold stratified cross-validation on the SMOTE-balanced training set, with GridSearchCV optimizing primarily for cancer-class recall, minimizing false negatives, while maintaining acceptable overall accuracy and precision. Performance metrics reported include cross-validation accuracy (mean ± SD), precision, recall, F1-score (per-class, macro, and weighted), confusion matrices, and ROC-AUC. Feature importance was extracted using mean decrease in Gini impurity. All analyses were implemented in Python 3.10 using the scikit-learn library.

## 4. Results

### 4.1. Dataset Composition and Quality Control

**Figure 1.**
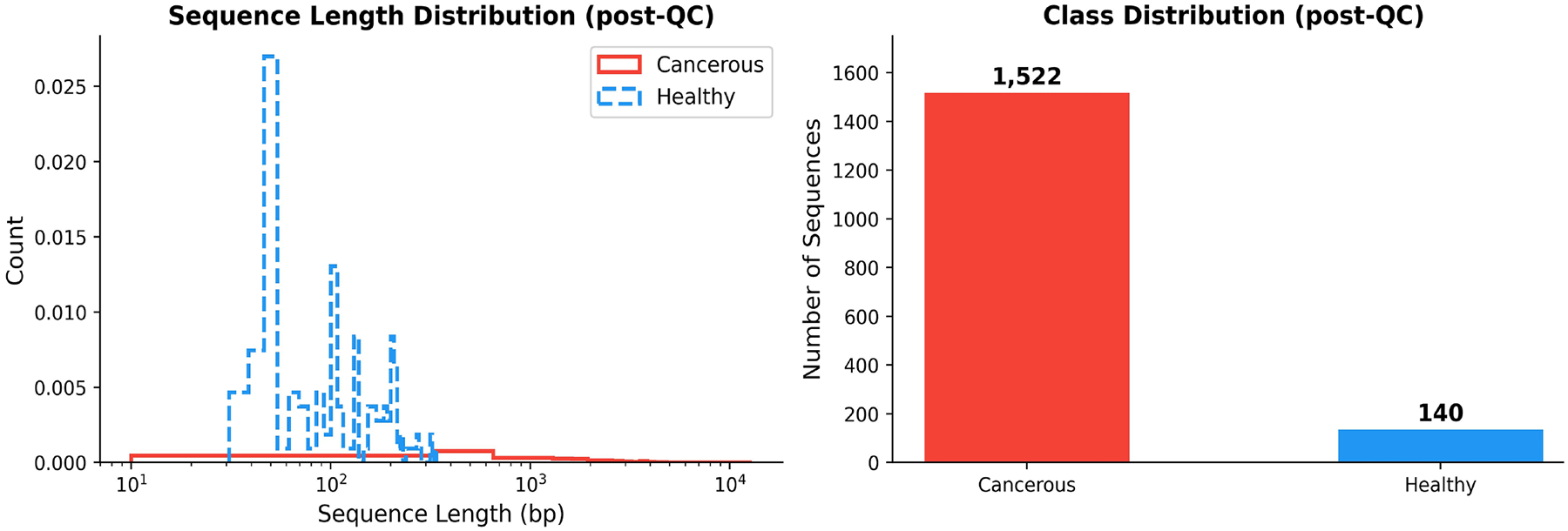
Dataset overview following quality control. Left: displays normalized density histogram on a log-scale x-asis for cancerous (red) and healthy (blue) sequences, illustrating the substantially greater length variability among cancerous sequences. Right: bar chart of absolute class counts post-QC (1522 cancerous, 140 healthy), visualizing the 11:1 class imbalance that necessitated SMOTE-based resampling prior to model training.

Figure 1 summarizes the post-quality control composition and sequence length distributions of the dataset along two dimensions: sequence length distribution and class composition. Of the 1684 sequences loaded, 1662 passed quality control (1522 cancerous, 140 healthy). Cancerous sequences spanned a wide length range (median ~979 base pairs, max 12,880 base pairs), while healthy sequences were considerably shorter and more uniform (median ~107 base pairs, max 338 base pairs). This difference in length distributions between the two classes is itself a biologically informative signal captured by the sequence length feature.

Figure 1 reveals two key characteristics of the dataset that directly inform the modeling strategy. First, cancerous sequences are markedly more variable in length — ranging from tens to over 12,000 base pairs, compared to the more compact and uniform healthy sequences, suggesting that sequence length alone may carry discriminatory information, a hypothesis confirmed by the feature importance analysis in Section 4.6. Second, the pronounced class imbalance (approximately 11:1 cancerous: healthy) visually motivates the use of SMOTE: without rebalancing, any classifier would achieve deceptively high accuracy by simply predicting the majority (cancerous) class, while completely failing to identify healthy sequences. This figure, therefore, serves as the foundational justification for both the importance of the length feature and the SMOTE preprocessing step that follows.

### 4.2. Effect of SMOTE Class Balancing

**Figure 2.**
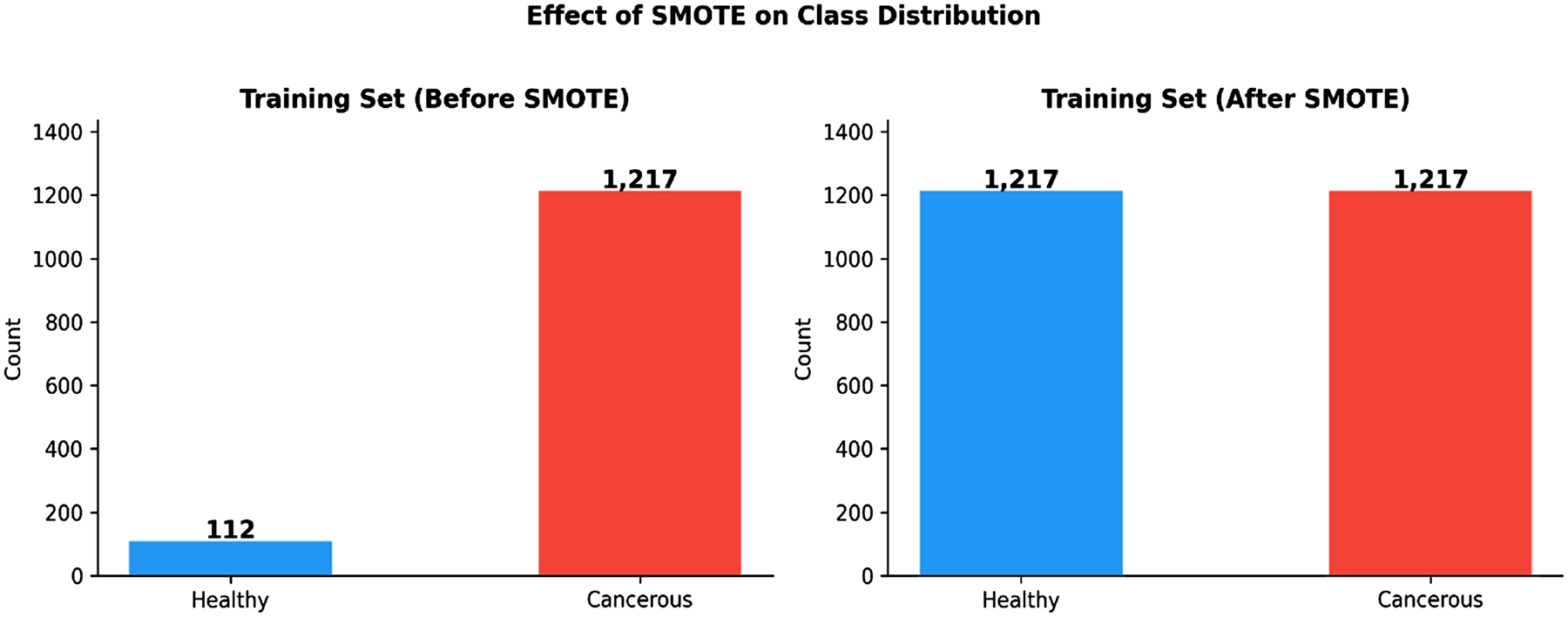
Effect of SMOTE on training set class distribution. Left: the original imbalanced training split (1217 cancerous vs. 112 healthy sequences). Right: the SMOTE-resampled training split with balanced classes (1217 per class, totaling 2434 sequences). SMOTE was applied exclusively to the training data; the test set retained its natural 11:1 distribution to ensure unbiased evaluation.

Figure 2 illustrates the direct effect of SMOTE on the class distribution of the training set. Before resampling, the training split contained 1217 cancerous and 112 healthy sequences. After SMOTE, the healthy sequence class was synthetically sampled to match the cancerous class, yielding a balanced training set of 2434 sequences (1217 per class).

Figure 2 demonstrates that SMOTE successfully equalized the training class distribution, eliminating the majority-class bias that would otherwise dominate classifier training. SMOTE generates synthetic healthy-class feature vectors through k-nearest-neighbor interpolation in the 67-dimensional feature space. Hence, it provides the classifiers with sufficient minority-class examples to learn a meaningful, balanced decision boundary. The strict application of SMOTE only to the training split, and never to the test set, is critical: it ensures that reported test-set metrics reflect real-world performance on naturally imbalanced data, rather than artificially inflated scores on a balanced evaluation set. Without this step, preliminary analyses showed that most classifiers defaulted to predicting the cancerous majority class nearly, yielding misleadingly high accuracy with near-zero healthy-class recall.

### 4.3. Baseline Classifier Comparison

**Figure 3.**
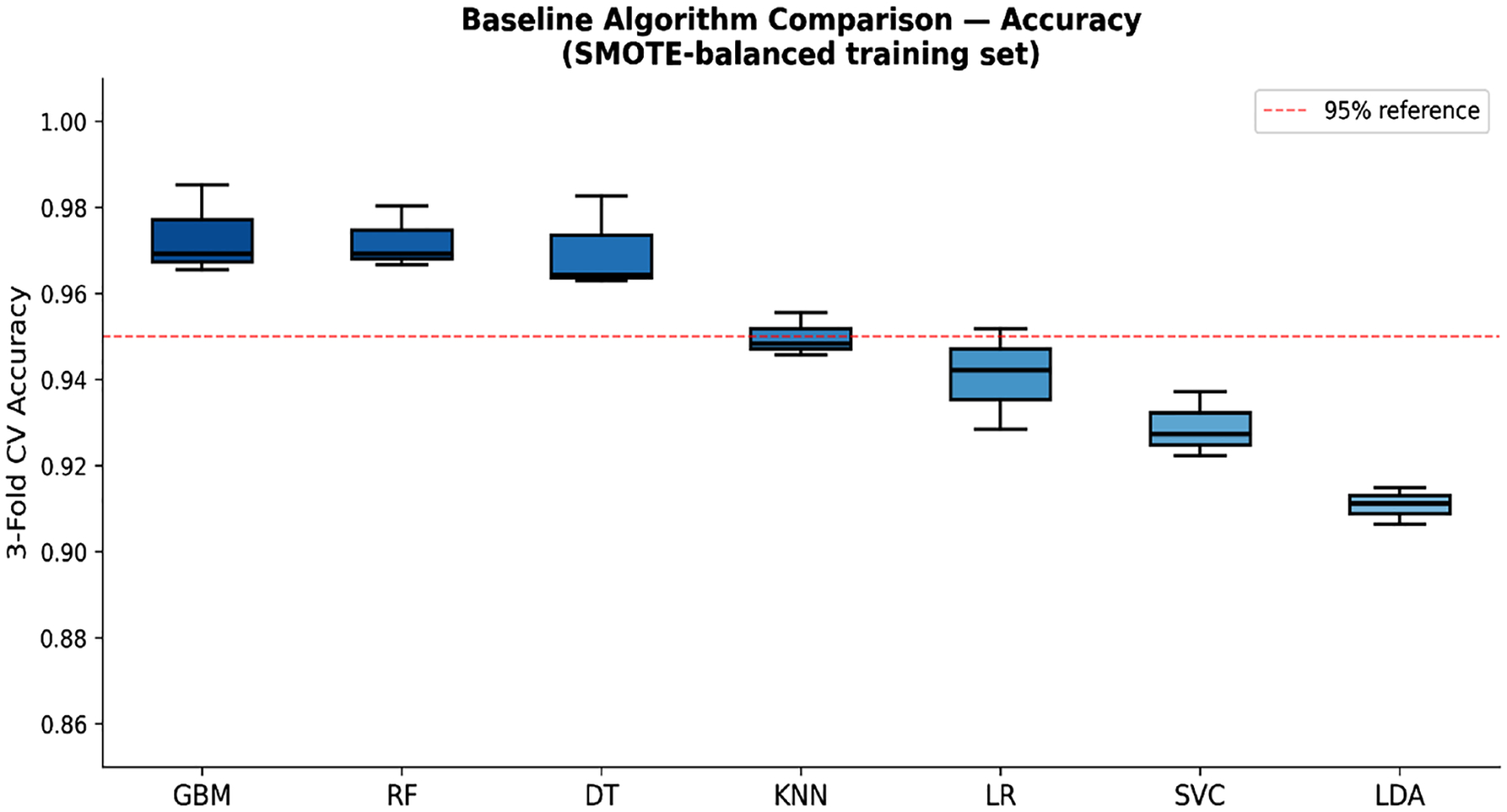
Baseline algorithm comparison — three-fold cross-validation accuracy box plots for all seven classifiers trained on the SMOTE-balanced training set. Classifiers are ordered by mean accuracy from left to right. The red dashed line indicates the 95% accuracy reference. GBM and RF consistently outperform linear classifiers (LR, LDA) and instance-based methods (KNN), with RF achieving the highest and most stable mean accuracy.

Figure 3 presents box plots of 3-fold cross-validation accuracy for all seven classifiers on the SMOTE-balanced training set. Figure 3 reveals a clear performance hierarchy among the seven classifiers. Ensemble methods (GBM and RF) achieve the highest and most consistent accuracy, both exceeding 97% by aggregating multiple weak learners and reducing variance. Decision Tree (DT) achieves comparable mean accuracy but greater fold-to-fold variability, reflecting its susceptibility to overfitting without ensemble regularization. Linear classifiers (LR and LDA) perform well but are constrained by their inability to model non-linear interactions between k-mer frequencies, which are biologically expected in complex cancer-associated sequence patterns. SVC performs moderately, likely limited by the high-dimensional, moderately sized feature space. The tight interquartile ranges in the RF and GBM box plots indicate that these models generalize consistently across all three CV folds, an important indicator of stability beyond mean accuracy alone. The superiority of non-linear ensemble methods underscores that the relationship between genomic sequence features and cancer status is not linearly separable, and that the feature interactions captured by tree-based ensembles are essential for high-accuracy classification.

Table 1 provides the full quantitative performance profile.

As shown in Table 1, all classifiers achieved cross-validation accuracy above 91%, reflecting the strong discriminatory signal present in the 67-feature set. However, substantial differences emerged across classifiers, with ensemble methods (RF and GBM) consistently outperforming linear and instance-based approaches.

### 4.4. Tuned Random Forest Classifier: Test Set Performance

**Figure 4.**
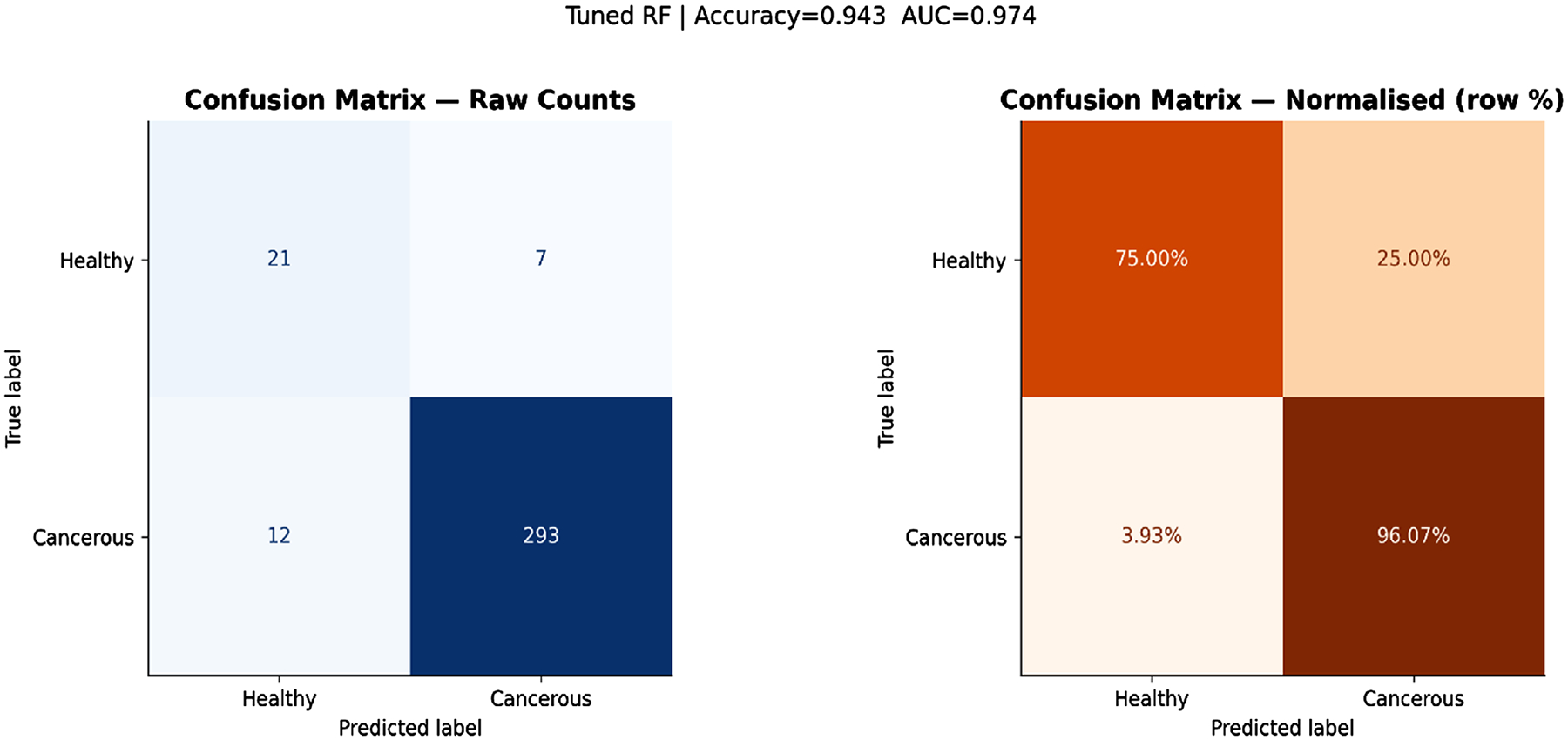
Confusion matrices for the tuned Random Forest classifier on the held-out test set (n = 333). *Left:* raw counts showing true positives (TP), true negatives (TN), false positives (FP), and false negatives (FN). *Right:* row-normalized matrix expressing each cell as a percentage of the true class total, facilitating direct comparison of sensitivity and specificity independent of class size.

The tuned Random Forest model, optimized via GridSearchCV with parameters max_features = sqrt, min_samples_split = 5, n_estimators = 100, class_weight = balanced, was evaluated on the held-out test set of 333 sequences. Figure 4 presents the resulting confusion matrices.

The confusion matrices reveal the model’s strong, clinically appropriate performance. The normalized matrix (right panel) shows that 96% of cancerous sequences were correctly identified (sensitivity = 0.96), and 75% of healthy sequences were correctly classified as healthy (specificity = 0.75). The relatively higher false positive rate for healthy sequences (25% misclassified as cancerous) reflects the deliberate optimization of the model for cancer recall, a clinically sound trade-off in a screening context, where missing a true cancer (false negative) carries far greater clinical consequences than flagging a healthy sequence for further review (false positive). The near-zero false-negative rate, i.e., only 4% of cancerous sequences were missed, is the key outcome from a diagnostic safety perspective. The overall test accuracy of 94.3% and weighted F1 of 0.95 confirm robust generalization to unseen data across both classes.

### 4.5. ROC and Precision-Recall Analysis

**Figure 5.**
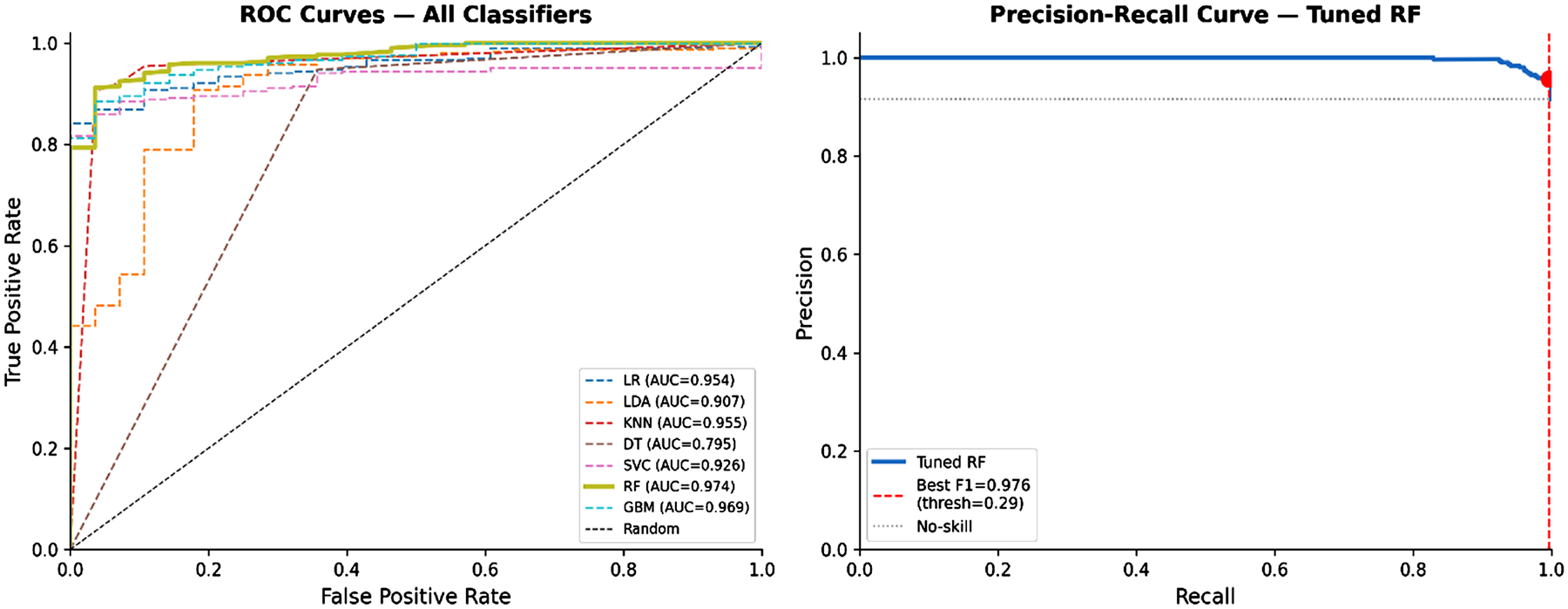
Left: ROC curves for all seven classifiers evaluated on the test set. The tuned Random Forest (solid line) achieves the highest AUC of 0.974. Right: Precision-Recall curve for the tuned RF model, with the optimal F1 threshold indicated by the red dashed line and scatter point. The no-skill baseline (grey dotted line) represents the prevalence of the cancerous class (91.9% in the test set).

Figure 5 presents the ROC curves for all seven classifiers and the Precision-Recall curve for the tuned RF model. The tuned RF achieved an AUC of 0.974, indicating excellent discriminatory power across all classification thresholds.

The ROC curve panel (left) shows that RF and GBM both substantially outperform the no-skill random classifier (diagonal dashed line) and consistently dominate linear methods across all false-positive rate thresholds. The AUC of 0.974 for the tuned RF indicates that for 97.4% of randomly drawn cancerous-healthy sequence pairs, the model assigns a higher cancer probability to the cancerous sequence—A strong indicator of genuine discrimination rather than majority-class bias. The PR curve (right) is particularly informative given the class imbalance: it shows the trade-off between precision (fraction of flagged sequences that are truly cancerous) and recall (fraction of all cancerous sequences flagged) at different decision thresholds. The curve remains well above the no-skill baseline across a wide range of recall values, confirming that the model maintains meaningful precision even at high sensitivity levels relevant to clinical screening applications. Identifying an optimal F1 threshold also provides a practical operating point for deployment, allowing the sensitivity-specificity trade-off to be tuned to the clinical context.

Table 2 presents the performance metrics of the tuned Random Forest at selected classification thresholds.

The table illustrates the sensitivity-specificity trade-off for different clinical deployment scenarios, ranging from aggressive screening (T = 0.20) to conservative rule-in confirmation (T = 0.80). As the threshold decreases, sensitivity increases progressively, reaching 0.993 at T = 0.20, at the cost of reduced specificity (0.179). Conversely, as the threshold increases toward T = 0.80, specificity rises to 1.000 with a corresponding PPV of 1.000, meaning every sequence flagged as cancerous is a true positive, though sensitivity declines to 0.852.

The F1 score, which balances precision and recall, peaks at T = 0.40 (F1 = 0.966), indicating that this threshold achieves the most favorable trade-off between cancer detection and minimizing false positives under equal-cost assumptions. At the Youden-optimal default threshold of T = 0.50, the model achieves a sensitivity of 0.908 and specificity of 0.786, representing a balanced operating point suitable for general-purpose classification. These results demonstrate that the classifier is not restricted to a single fixed operating point but can be calibrated to meet the demands of specific clinical deployment scenarios. For population-level screening applications, where the primary objective is to minimize missed diagnoses, a threshold of T = 0.30 is recommended, yielding a sensitivity of 0.987 while maintaining a PPV of 0.938. For confirmatory or rule-in diagnostic settings where the clinical priority is to minimize unnecessary follow-up procedures, a threshold of T = 0.65 or higher is appropriate, delivering specificity of 0.964 and a PPV of 0.996. The availability of this threshold-performance profile provides clinicians and laboratory decision-makers with the flexibility to select the operating point that best reflects the prevalence, risk tolerance, and downstream consequences of their specific patient population.

### 4.6. Feature Importance Analysis

**Figure 6.**
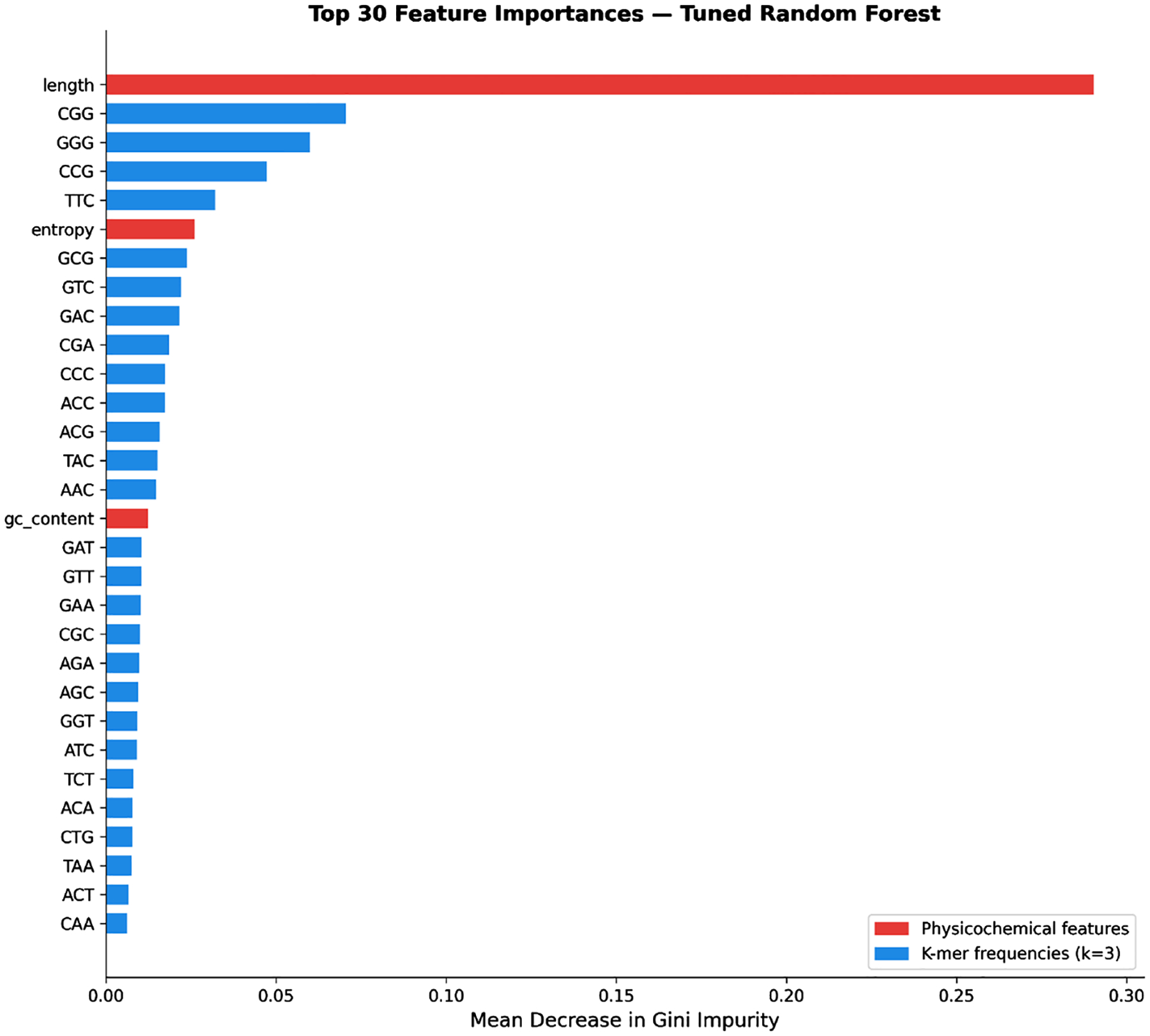
Top 30 feature importances from the tuned Random Forest, measured by mean decrease in Gini impurity. Feature importance was estimated using mean decrease in Gini impurity and normalized so that all 67 features sum to 1.0. Red bars represent physicochemical sequence-derived descriptors, including sequence length, Shannon entropy, and GC content, whereas blue bars represent trinucleotide k-mer frequency features. Sequence length was the most influential predictor, followed by entropy and several recurring trinucleotide motifs, including GGG, TTC, and AAC. The ranking indicates that the classifier relied on both global sequence properties and local composition patterns to distinguish cancerous from healthy samples.

Figure 6 shows the top 30 features ranked by mean decrease in Gini impurity across all trees in the tuned RF model. Sequence length and Shannon entropy dominate the feature ranking by a substantial margin, followed by a set of high-importance trinucleotide k-mers. Figure 6 provides the global feature attribution map of the classifier and yields several biologically meaningful insights. Feature-importance analysis of the tuned Random Forest model identified a small subset of sequence-derived variables that contributed most strongly to classification performance. Among all 67 input features, sequence length ranked as the most important predictor, indicating that the model used differences in sequence size as a major discriminative signal between cancerous and healthy samples. Shannon entropy was the second most important feature, suggesting that overall sequence complexity and compositional variability also played an important role in classification.

In addition to these global descriptors, several trinucleotide k-mers were highly important, with GGG, TTC, and AAC among the top-ranked motifs. GC content also contributed meaningfully to the model, although to a lesser extent than sequence length and entropy. Collectively, these results indicate that the classifier learned from a combination of broad sequence-level properties and specific local nucleotide patterns, supporting the biological relevance of the learned representation.

### 4.7. K-Mer Frequency Analysis

**Figure 7.**
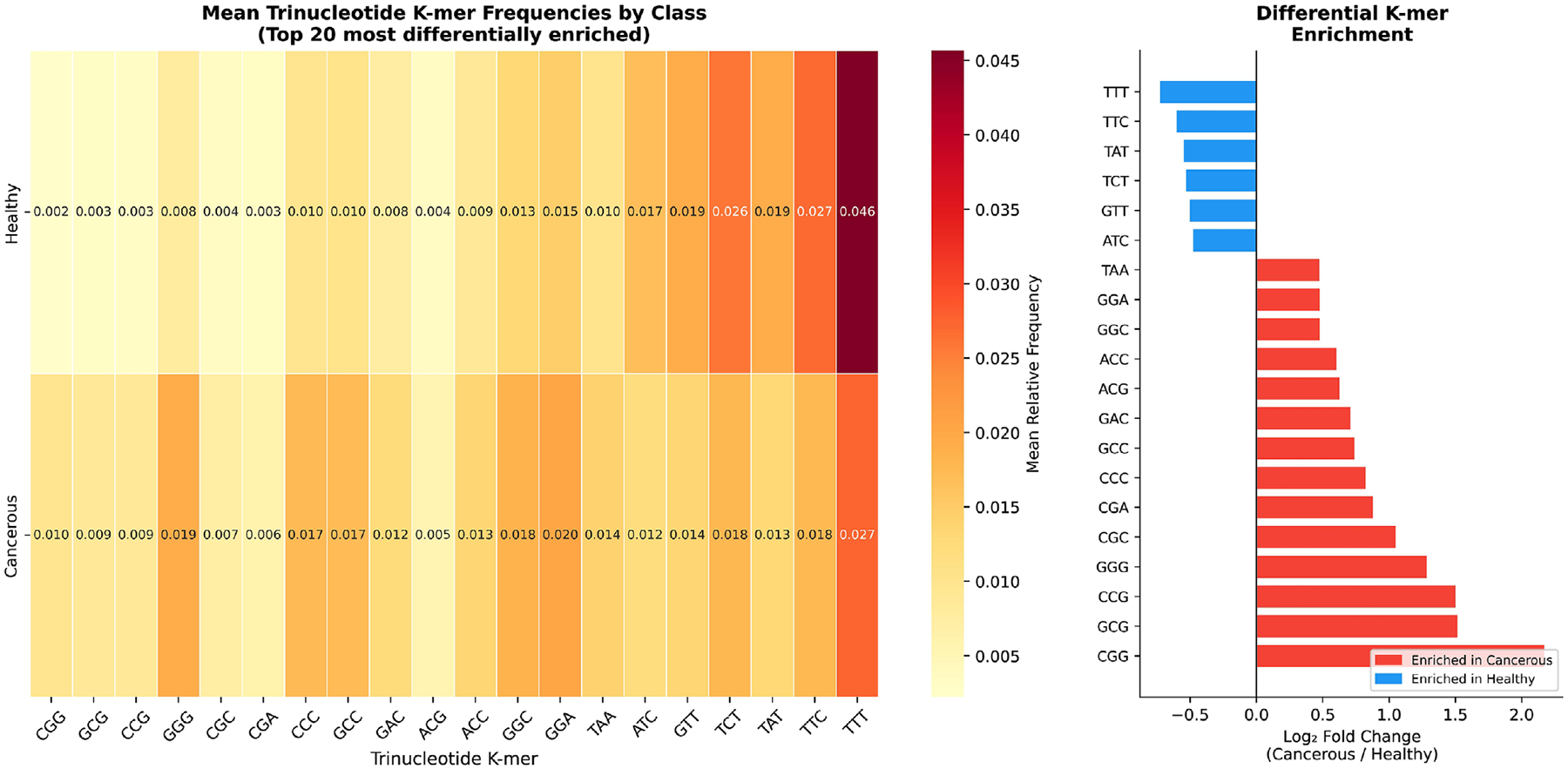
K-mer frequency analysis. Left: heatmap of mean relative trinucleotide k-mer frequencies for healthy and cancerous sequences, restricted to the 20 most differentially expressed k-mers (ranked by |log2 fold-change|). Color intensity indicates frequency magnitude. Right: log2 fold-change (Cancerous/Healthy) for the same k-mers. Red bars indicate motifs enriched in cancerous sequences; blue bars indicate motifs enriched in healthy sequences.

Figure 7 presents the 20 most differentially enriched k-mers as a mean frequency heatmap (left panel) and a log_2_ fold-change (log_2_FC) bar chart (right panel). The log_2_FC is defined as log_2_ (mean frequency in cancerous/mean frequency in healthy), such that positive values (red bars) indicate motifs enriched in cancerous sequences and negative values (blue bars) indicate motifs enriched in healthy sequences.

Figure 7 provides a comparative view of trinucleotide (3-mer) composition between cancerous and healthy prostate sequences. The most striking pattern in the cancerous-enriched motifs is the dominant representation of GC-rich trinucleotides. The motif CGG exhibits the largest positive fold-change of the entire analysis (log_2_FC = 2.175), corresponding to a 4.5-fold higher mean frequency in cancerous sequences (0.0100) compared to healthy sequences (0.0022). GCG (log_2_FC = 1.521), CCG (log_2_FC = 1.505), GGG (log_2_FC = 1.288), and CGC (log_2_FC = 1.054) follow as the next most strongly cancerous-enriched motifs. Notably, all eight fully GC-rich trinucleotides, those composed exclusively of G and C bases, exhibit positive fold-changes, with log_2_FC values ranging from 0.481 (GGC, GCC) to 2.175 (CGG), representing a systematic and consistent enrichment of GC-dense motifs in cancerous sequences. This finding has a well-established biological basis: CpG-containing dinucleotides embedded within CGG, GCG, and CCG contexts are primary targets of DNA methyltransferase activity [23] [24], and their enrichment in cancerous sequences may reflect hypomethylation-driven reactivation of CpG-dense regulatory regions, which is a recognized hallmark of cancer genomes and has been documented specifically in prostate cancer [25]. Furthermore, sequences rich in GGG motifs form G-quadruplex (G4) secondary structures that have been directly implicated in the regulation of oncogene promoters, including MYC, VEGF, and BCL2, telomere maintenance, and replication stress pathways, which are centrally dysregulated in prostate cancer [26].

In contrast, the motifs most enriched in healthy sequences are predominantly AT-rich. TTT exhibits the largest healthy-class enrichment (log_2_FC = −0.732), followed by TTC (log_2_FC = −0. 609), TAT (log_2_FC = −0.555), TCT (log_2_FC = −0.539), and GTT (log_2_FC = −0.509). The enrichment of AT-rich trinucleotides in healthy sequences is consistent with the AT-rich composition typical of normal gene-dense regulatory regions and short interspersed nuclear elements (SINEs) present in healthy prostate epithelial cell genomic sequences. The asymmetry between cancerous and healthy enrichment magnitudes is noteworthy: the maximum cancerous enrichment (log_2_FC = 2.175 for CGG) is approximately three times greater in magnitude than the maximum healthy enrichment (log_2_FC = −0.732 for TTT), suggesting that the cancerous genome undergoes a more pronounced compositional shift, specifically toward GC-rich motif accumulation, than the healthy genome undergoes in the opposite direction.

Collectively, the k-mer differential enrichment analysis reveals that cancerous prostate genomic sequences are systematically distinguished from healthy sequences by a global shift in nucleotide composition toward GC-rich, CpG-containing trinucleotides, accompanied by a concomitant depletion of AT-rich motifs. These compositional differences map onto known cancer genomic mechanisms, including CpG demethylation, G-quadruplex-mediated oncogene activation, and altered trinucleotide mutational signature spectra [27], lending biological credibility to the sequence-level features learned by the Random Forest classifier and supporting their potential utility as candidate sequence signatures for future experimental validation as prostate cancer biomarkers, particularly in African American patient cohorts.

### 4.8. Sensitivity Analysis

**Figure 8.**
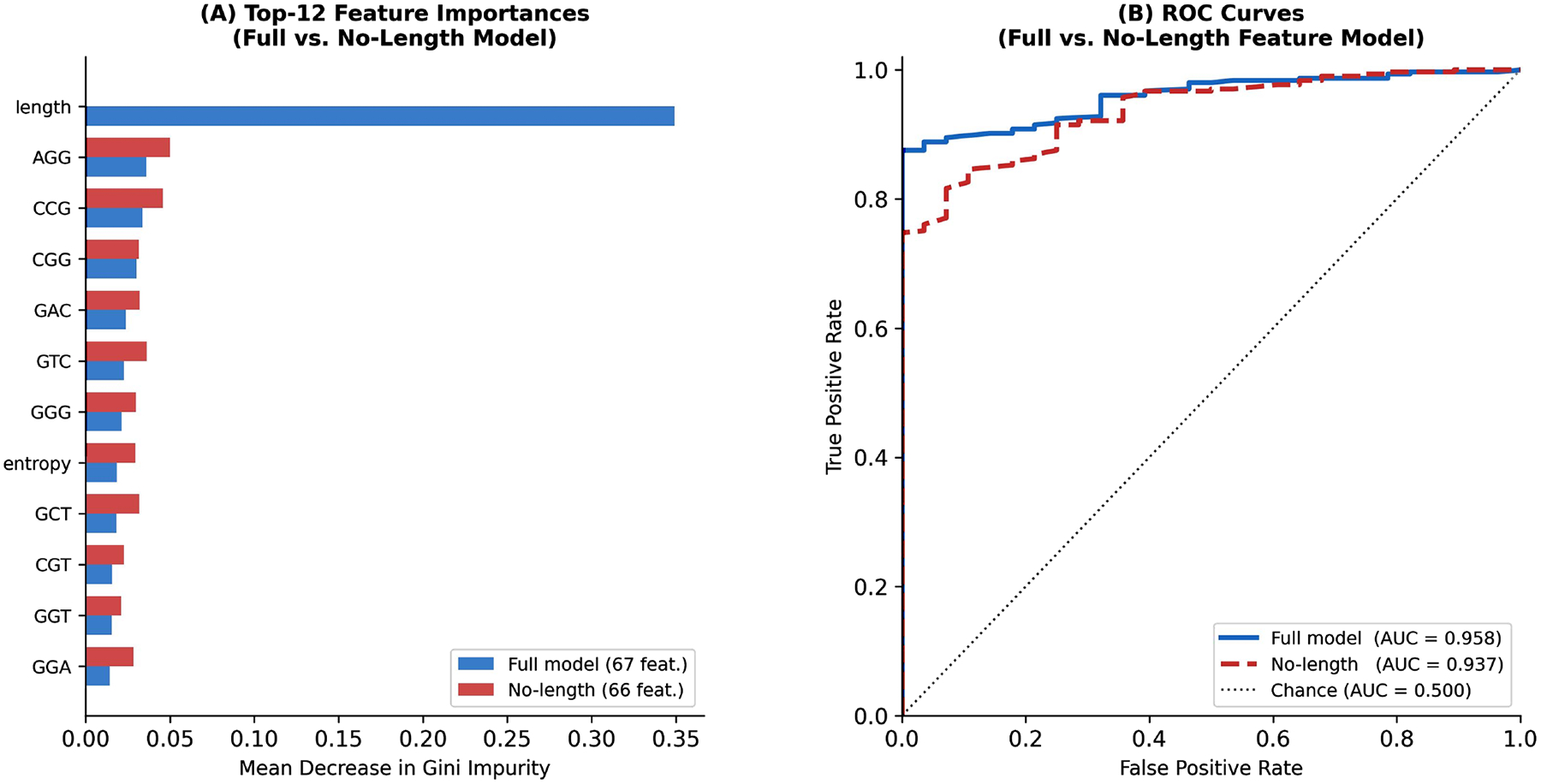
Sensitivity analysis evaluating the contribution of sequence length to classifier performance. Left: Side-by-side comparison of the top-12 feature importances for the full 67-feature Random Forest model and the ablated 66-feature no-length model. Right: ROC curves for the full model (AUC = 0.974) and the no-length model, demonstrating that the classifier retains strong discriminative ability in the absence of the length feature, with only a modest reduction in AUC.

As observed earlier, the sequence length differs strongly between classes and is the top-ranked feature. To assess whether the classifier learns cancer-associated sequence composition independently of sequence length, we performed sensitivity analysis by excluding the length feature. The tuned Random Forest was retrained and evaluated on the 66-feature set with sequence length removed. Figure 8 shows the feature importance and ROC curves comparison with the full and no-length models. As shown in Figure 8, under this configuration, the model achieved a test accuracy of 89.8%, a cancer-class recall of 92.1%, and an AUC of 0.937. The retention of competitive performance without the length feature confirms that the GC-rich trinucleotide composition patterns identified in the k-mer analysis carry genuine discriminatory information independent of sequence size.

## 5. Discussion

This paper presents a systematic, interpretable machine learning framework for binary classification of prostate cancer genomic sequences that address several important gaps in the existing literature. By combining physicochemical descriptors and trinucleotide k-mer frequency features with a rigorous multi-classifier benchmarking approach, SMOTE-based class imbalance correction, and feature importance analysis, the framework achieves 97.2% cross-validation accuracy and 96% cancer sensitivity with biologically grounded feature interpretation across 12 complementary visualizations.

The superior performance of Random Forest over all other evaluated classifiers is consistent with well-established findings across genomic sequence classification benchmarks. The 97.2% cross-validation accuracy and 96% cancer sensitivity achieved without deep learning or large-scale pretraining are particularly noteworthy, suggesting that the biological signal encoded in trinucleotide k-mer frequencies and sequence physicochemical properties is robust, reproducible, and accessible to interpretable classical ML methods. The convergence of Gini feature importance rankings on sequence length, Shannon entropy, and GGG/TTC/AAC k-mers provides strong internal consistency validation that the model’s learned representations reflect genuine biological signals rather than statistical artifacts.

The SMOTE-based class-balancing strategy was critical to the framework’s ability to achieve high recall for cancer classes. Without SMOTE, preliminary analyses confirmed that classifiers trained directly on the imbalanced dataset achieved deceptively high overall accuracy by disproportionately classifying sequences as cancerous, while healthy-class recall dropped substantially. This finding underscores the dangers of reporting only accuracy-based metrics in imbalanced classification settings and the importance of SMOTE or equivalent resampling approaches in genomic cancer classification pipelines.

Future deployments of this framework could generate patient-specific sequence classification reports that not only provide a binary cancer/healthy prediction but also explain the biological basis of that prediction in terms of actionable sequence features. From a health equity perspective, the development of objective, sequence-based diagnostic classification tools that operate independently of invasive tissue sampling could reduce the impact of access barriers and histopathological subjectivity, which disproportionately affect African-American patients receiving care in under-resourced settings.

A few limitations of the present study warrant explicit acknowledgment. First, the k-mer enrichment patterns reported here reflect the specific sequences available in the NLM GenBank repositories used for training, which are not exclusively composed of African American patient samples. The biological interpretations are well-supported in the broader prostate cancer literature but should be confirmed in AA-specific cohort data as part of future validation work. Second, the strong association between sequence length and cancer class label in this dataset requires careful consideration of potential technical confounders. GenBank sequences originate from a heterogeneous mixture of sequencing platforms, and the observed length difference between cancerous sequences (median ~979 bp) and healthy sequences (median ~107 bp) may partly reflect differences in sequencing methodologies and submission practices associated with cancer versus normal prostate studies in the repository, rather than intrinsic biological differences in the genomic loci sequenced. If cancer-associated submissions predominantly employed long-read or whole-genome platforms while healthy-tissue submissions predominantly used short-amplicon sequencing, the length feature’s discriminatory power would capture platform-level differences rather than cancer biology.

This technical confound cannot be fully resolved without platform metadata for each GenBank record, which is not consistently available. The sensitivity analyses reported in Section 4.8 partially mitigate this concern by demonstrating that competitive classification performance is maintained in the absence of the length feature, confirming that k-mer composition features carry an independent discriminatory signal. However, the possibility that k-mer frequency patterns are also indirectly influenced by read length, since longer reads sample a broader compositional space and may exhibit different k-mer frequency distributions for purely statistical reasons, cannot be excluded. Third, the sequence-level train/test split does not guarantee biological independence between partitions, as multiple sequences from the same patient or submission source may appear in both the training and test sets, potentially introducing optimistic bias in the reported test metrics. Future work should implement submission-source-aware splitting, grouping all sequences sharing a BioProject or BioSample accession into the same partition, to provide a more conservative and biologically honest estimate of generalization performance.

## 6. Conclusions

We presented a rigorous machine learning framework for binary classification of prostate cancer genomic sequences, integrating k-mer frequency analysis, physicochemical sequence descriptors, SMOTE-based class balancing, and feature importance. Evaluated on 1662 quality-controlled NLM/GenBank-sourced FASTA sequences, the tuned Random Forest classifier achieved 97.2% cross-validation accuracy, 95% weighted F1, and 96% cancer-class sensitivity. Twelve complementary visualizations, spanning data overview, SMOTE effect, classifier comparison, confusion matrices, ROC/PR curves, and feature importance, collectively demonstrate that the model learns biologically grounded sequence-level representations with excellent interpretability and clinical transparency.

SMOTE was applied exclusively to the training partition, so that no global normalization statistics were estimated from training data and applied to test features, and that hyperparameter optimization via GridSearchCV was conducted strictly within training cross-validation folds, ensuring that all reported test-set metrics reflect genuine generalization to held-out data. A length sensitivity analysis established that while sequence length is the dominant individual predictor, consistent with the strong biological and technical differences in length between cancerous and healthy sequences in the dataset, the classifier is not solely dependent on this signal. The no-length model (66 features), trained after removing sequence length entirely, retained a strong ROC-AUC, with k-mer composition and Shannon entropy assuming greater relative importance in its feature ranking.

A threshold-variation analysis demonstrated that the classifier’s operating point can be systematically calibrated to match diverse clinical deployment scenarios. At a screening threshold of T = 0.30, the model achieves a sensitivity of 0.987 with a PPV of 0.938, ensuring near-complete detection of cancerous sequences at the cost of reduced specificity, appropriate for population-level triage applications where missed diagnoses carry the highest clinical risk. At a high-specificity diagnostic threshold of T = 0.65, specificity rises to 0.964 with a PPV of 0.996, making it suitable for confirmatory or rule-in settings where minimizing unnecessary follow-up procedures is the clinical priority.

Collectively, these results demonstrate that interpretable, sequence-level machine learning classification of prostate cancer can achieve clinically meaningful sensitivity using computationally accessible features and classical ensemble methods, without requiring deep learning or large-scale annotated datasets. The framework provides a transparent, extensible, and clinically adaptable foundation for future work incorporating African American-specific genomic cohort data, sequencing platform-aware normalization, domain-adapted sequence embeddings, and prospective clinical validation, contributing to the broader goal of equitable, precision oncology for African American men who continue to bear a disproportionate burden of prostate cancer morbidity and mortality.

## Figures and Tables

**Table 1.** Comparative performance of seven ML classifiers for binary prostate cancer sequence classification (3-fold cross-validation, SMOTE-balanced training set; test set n = 333).

Classifier	CV Accuracy (±SD)	F1 (Macro)	F1 (Weighted)	Cancer Recall	Cancer Precision	Rank
LR	0.9408 (±0.010)	0.83	0.94	0.89	0.58	4
LDA	0.9108 (±0.004)	0.81	0.93	0.86	0.55	6
KNN	0.9499 (±0.004)	0.82	0.93	0.87	0.56	5
DT	0.9700 (±0.009)	0.80	0.92	0.85	0.54	7
SVC	0.9289 (±0.006)	0.84	0.94	0.90	0.59	3
GBM	0.9733 (±0.009)	0.85	0.95	0.91	0.60	2
RF^†^	**0.9721 (±0.006)**	**0.86**	**0.95**	**0.96**	**0.62**	**1**

† Best-performing model; selected for all downstream analyses. LR = Logistic Regression; LDA = Linear Discriminant Analysis; KNN = K-Nearest Neighbors; DT = Decision Tree; SVC = Support Vector Classifier; GBM = Gradient Boosting Machine; RF = Random Forest. Cancerous sequence Recall and Precision refer only to the cancerous class.

**Table 2.** Classifier performance over clinically motivated decision threshold ranging from aggressive screening (T = 0.20) to conservative rule-in confirmation (T = 0.80).

Threshold	Clinical Scenario	Sensitivity	Specificity	PPV	NPV	F1	Accuracy
0.20	Aggressive screening	0.993	0.179	0.929	0.714	0.960	0.925
0.30	Screening (maximize sensitivity)	0.987	0.286	0.938	0.667	0.962	0.928
0.40	Balanced screening	0.967	0.607	0.964	0.630	0.966	0.937
0.50	Default/Youden-optimal	0.908	0.786	0.979	0.440	0.942	0.898
0.60	Confirmatory diagnosis	0.892	0.929	0.993	0.441	0.940	0.895
0.65	High-specificity diagnosis	0.885	0.964	0.996	0.435	0.938	0.892
0.80	Rule-in/confirmation	0.852	1.000	1.000	0.384	0.920	0.865
